# Obtundents for Sensitive Dentine

**Published:** 1894-08

**Authors:** 


### Obtundent for Sensitive Dentine.

| | | |
|---|---|---|
| R. | Cocaine hydrochlor | 5 grains |
| | Chloroformi | ½ drachm |
| | Acid. hydrochlor | 10 minims |
| | Alcohol | ½ drachm |
| Mix. | | |

